# A Nutrition Education Intervention Using NOVA Is More Effective Than MyPlate Alone: A Proof-of-Concept Randomized Controlled Trial

**DOI:** 10.3390/nu11122965

**Published:** 2019-12-06

**Authors:** Aydin Nazmi, Marilyn Tseng, Derrick Robinson, Dawn Neill, John Walker

**Affiliations:** 1 Food Science and Nutrition Department, California Polytechnic State University, San Luis Obispo, CA 93407, USA; 2 Kinesiology and Public Health Department, California Polytechnic State University, San Luis Obispo, CA 93407, USA; mtseng@calpoly.edu; 3 University of California, Division of Agriculture and Natural Resources, Davis, CA 95618, USA; 4 Interdisciplinary Studies in the Liberal Arts, California Polytechnic State University, San Luis Obispo, CA 93407, USA; dbneill@calpoly.edu; 5 Statistics Department, California Polytechnic State University, San Luis Obispo, CA 93407, USA; jwalker@calpoly.edu

**Keywords:** NOVA food classification, MyPlate, US Dietary Guidelines, nutrition education, food literacy, diet, food, nutrition

## Abstract

The ability to classify foods based on level of processing, not only conventional MyPlate food groups, might be a useful tool for consumers faced with a wide array of highly processed food products of varying nutritional value. The objective of this study was to assess the impact of a proof-of-concept nutrition education intervention on nutrition knowledge, assessed by correct classification of foods according to MyPlate food groups, MyPlate ‘limit’ status (for fat, sugar, sodium), and level of processing (NOVA categories). We utilized a randomized, controlled design to examine the impact of a MyPlate vs. combined MyPlate + NOVA intervention vs. control group. Intervention groups received educational flyers via email and participants were assessed using electronic baseline and follow-up surveys. The MyPlate + NOVA intervention group performed at least as well as the MyPlate group on classifying conventional food groups and ‘limit’ status. Moreover, the MyPlate + NOVA group far outperformed the other groups on classifying NOVA categories. Longer-term trials are needed, but our results suggest that NOVA principles may be more easily understood and applied than those of MyPlate. Education strategies focusing on level of food processing may be effective in the context of the modern food environment.

## 1. Introduction

Nutrition-related chronic diseases represent a massive and largely preventable public health [1,2] and economic [3,4] burden in the United States, but the effectiveness of nutrition education strategies has been limited [5,6].

The US Dietary Guidelines defines five food groups (fruits, vegetables, grains, proteins, dairy) and suggests limiting added sugars, sodium, and fats [7]. MyPlate, an educational component of the Guidelines, “…help(s) consumers make better food choices [8]”. Nevertheless, the American diet remains notoriously unhealthy. Data from the Behavioral Risk Factor Surveillance System indicate that 76% and 87% of adults, respectively, do not meet recommendations for fruit and vegetable [9] intake and 90% consume excessive sodium [10]. More than half drink sugar-sweetened beverages at least daily [11].

A limitation of the Guidelines and MyPlate is that they do not address the range of choices available in the modern American food environment. Many foods are not easily classified because they are combination foods (e.g., pizza) or because classification is not straightforward, (e.g., sweet potato chips, fruit leather). Another limitation is that there is no guidance on how to distinguish foods with excessive amounts of saturated fat, added sugars, and sodium from foods that do not. For example, ready-to-eat high-sugar breakfast cereals and plain oatmeal are both categorized as ‘grains’. They fall within the same food group and may lead consumers to believe that these foods are nutritionally equal, when in fact, the former contains far less grain, derives much of its calories from added sugars, and contains a high amount of sodium. Delivering information that helps consumers clearly identify foods that should be limited would be a useful supplement to dietary guidelines.

Another aspect of the modern American food environment that is unaddressed by the Dietary Guidelines is the ubiquity of ultra-processed food products [12,13]. According to nationally representative data, 60% of calories consumed by Americans come from “ultra-processed” foods [14]. Ultra-processed foods have poor nutritional profiles, including more sugars, sodium, and fats [14,15,16,17,18,19]. International data have shown strong links between consumption of ultra-processed foods and obesity [20,21,22], hypertension [23], and metabolic syndrome [24]. Thus, recent food classification systems focus on extent of food processing rather than solely on nutrient content [25]. The most utilized in the literature is NOVA, which designates four groups: (1) unprocessed or minimally processed foods, (2) processed culinary ingredients, (3) processed foods, and (4) ultra-processed food and drink products. Dietary guidelines in several countries discourage consumption of ultra-processed foods, but in the US, guidance is conspicuously absent in the face of their widespread availability and accessibility. The ability to classify foods based on level of processing, not just on food groups, might be a useful tool for consumers faced with a wide array of food products of varying nutritional value.

The objective of this study was to assess the impact of a proof-of-concept nutrition education intervention on nutrition knowledge among college students. Offering alternative nutritional education opportunities focused on identifying and classifying the nutritional information of highly processed food is especially important for youth/young adults, who have the lowest consumer expenditure proportion for food at home and the highest away from home (www.bls.gov/cex). Food purchased for consumption away from home is linked to lower dietary quality, especially for those 16–24 years old [26]. Therefore, examining alternative nutrition or food education interventions among this cohort could bring the greatest benefit for improving overall dietary quality in the population.

We hypothesized that compared with those receiving no nutrition education, (1) participants who received MyPlate education would show a greater improvement in their ability to classify foods into MyPlate food groups and ‘limit’ categories and (2) participants who received additional education on NOVA would show greater improvement in their ability to classify foods by level of processing. A secondary hypothesis was that NOVA education would improve participants’ ability to identify ‘limit’ foods.

## 2. Materials and Methods

A total of 253 undergraduate students enrolled in three general education courses at a large public university were invited to participate in this randomized, controlled trial via email. Students were assigned to take part in this activity as part of their course, but were given the option to have their data included in the research study. Women made up 52% of the sample. Other social and demographic variables were not collected but were estimated to be in line with university demographics (mean age 20 years, 59% White/15% Latino/12% Asian/7% multi-racial/ <1% African-American). They were randomly assigned to one of three intervention groups: US Dietary Guidelines MyPlate intervention (MyPlate), MyPlate plus NOVA intervention (MyPlate + NOVA), and a control group that received no intervention.

### 2.1. Intervention

The MyPlate intervention consisted of a one-page educational flyer with language and visuals directly from the 2015 to 2020 US Dietary Guidelines [7]. The NOVA intervention included one additional page of images, narrative, and examples representing each NOVA group and the NOVA ‘golden rule’ (always prefer minimally processed foods and meals to ultra-processed food products). The intervention materials are available upon request.

### 2.2. Survey

A link to the baseline electronic survey was delivered via email. The survey contained three questions for each of 25 foods (Table 1) that represented each MyPlate food group and some combination foods, all NOVA categories, home-cooked and industrially-prepared versions of similar foods, and common foods that would be identifiable by young adults. The questions were as follows: (1) “In which MyPlate category does this food belong?” (Response options: fruits, vegetables, grains, protein, dairy, none of the above, (check all that apply), or I don’t know, (2) “Is this food high in any dietary components that the Dietary Guidelines say you should limit consuming?” (Response options: yes, no, I don’t know), and (3) In which NOVA category does this food belong?” (Response options: unprocessed/minimally processed, processed culinary ingredients, processed, ultra-processed, I don’t know). NOVA constructs were included in the survey to all participants, even those who did not receive the combined MyPlate + NOVA intervention, as participants may have had pre-existing knowledge of “ultra-processed” foods stemming from the ample popular press and media coverage of NOVA principles.

The online survey remained open for one week, with reminders sent on days 3 and 4, and closed on the seventh day. On the first day of the second week, the one- or two-page (MyPlate or MyPlate + NOVA, respectively) educational intervention was delivered electronically, which participants were instructed to study. This was followed in the same day by an email link to an identical follow-up survey containing the same questions as baseline, utilizing the same timeline.

The three outcomes of interest were correct classification of (1) MyPlate food groups, (2) MyPlate ‘limit’ status, and (3) NOVA food categories. Correct responses for MyPlate food groups and ‘limit’ status were determined using the USDA SuperTracker’s Food-A-Pedia (now discontinued), selecting the closest possible food item match from the options within the database. NOVA food categories were determined by authors through discussion according to previously published literature [27,28].

### 2.3. Statistical Analysis

Mean proportion correct and incorrect, percentage point difference between baseline and follow-up, and *p*-value by paired t-test was calculated for each of the three questions across each of the 25 foods by treatment group. McNemar’s Test is a statistical procedure for testing differences in binary data before and after an intervention [29]. For an individual subject, the test tracks the number of food items for which classification has changed positively (from incorrect to correct) and negatively (from correct to incorrect) after the intervention. For each subject *i*, we calculate a separate test statistic, *M_i_*, using McNemar’s Test using the R software package (Version 3.6.1). The significance level was set at 5%.

(1) $$M_{i}=\frac{\left(a_{i}-b_{i}\right)}{\sqrt{a_{i}+b_{i}}}$$

where *a_i_* is the number of food items that changed classification positively after intervention and *b_i_* is the number of food items that changed classification negatively after intervention. An individual’s McNemar statistic, *M_i_*, will be positive if more items change classification positively than negatively. The statistic will be negative if more items change classification negatively than positively. Items with classifications that do not change after intervention are not counted in the test statistic.

After computing McNemar statistics for each individual for each of the 25 foods, these statistics were used as the response variables in three separate analysis of variance (ANOVA) models—one for each classification question (MyPlate food classification, MyPlate limit status classification, and NOVA classification). The factors in these models were the intervention group, sex, and in which of the three general education classes the student was enrolled. A possible interaction between intervention group and sex was included to see if any intervention effects differed by sex. Observations in the ANOVA were given different weights based on the total number of classifications $\left(a_{i}+b_{i}\right)$ that changed for that individual after the intervention. The ANOVA calculations were done using the JMP software program (Version 14.3). Due to the large number of factors tested in the ANOVA, tests were evaluated at the 1% significance level to reduce the overall chance of Type I error across all analysis. For factors with statistically significant differences, follow-up pairwise comparisons were conducted using Tukey’s method.

### 2.4. Institutional Review Board

All elements of this research were approved by the Institutional Review Board at California Polytechnic State University, San Luis Obispo. Electronic informed consent was collected.

## 3. Results

Of the 253 invited to participate, 207 responded to baseline and were invited to also participate in follow-up, to which 181 responded. Complete data were available for 174 participants (69% participation rate).

Table 2 shows mean proportion (SD) of correct responses at baseline and follow-up for each of the three survey questions (averaged over all 25 foods) and percentage point difference (95% CI) in mean correct responses from baseline to follow-up. The MyPlate intervention group improved significantly only for the MyPlate limit status question, showing a 3.6 percentage point increase (95% CI 0.6, 6.7). The MyPlate + NOVA group improved significantly across all three questions, with increases of 4.0 (95% CI 1.0, 7.0), 6.0 (95% CI 3.2, 9.0), and 11.1 percentage points (95% CI 6.8, 15.3) for the three survey questions, respectively. Performance in the control group was mixed, with changes in opposite directions for the MyPlate food group and NOVA questions.

Table 3 shows the *p*-values for the F-tests of each factor in the three different ANOVA models. *p*-values less than 0.01 indicate statistical significance. In each ANOVA reported, a small number of subjects (no more than three) were excluded because they had no changes in classification following the intervention. For these individuals, the McNemar’s statistic was undefined since $\left(a_{i}+b_{i}\right)=0$.

There were statistically significant differences in the average McNemar statistic between the intervention groups for all three classification questions, indicating significant differences in the number of responses that changed from baseline to follow-up between intervention groups. Differences by class were also detected for both the MyPlate food group classification and the NOVA classification questions.

Table 4 shows the mean McNemar statistic, M, for each intervention group in the three different classification question ANOVA models. Positive means indicate that identification was better on average after the intervention. Negative means indicate that identification was worse on average after identification. For each classification question, means with different superscripts showed statistically significant differences using Tukey’s pairwise comparisons at the 1% significance level.

For the MyPlate food group question, the MyPlate + NOVA intervention had significantly better classification than the control group (average M = 0.50 and −0.51, respectively), but the differences between the MyPlate + NOVA group and the MyPlate group (average M = 0.50 and 0.19, respectively) were not detectable at the 0.01 alpha level. For the MyPlate limit status classification question, both the MyPlate + NOVA and MyPlate interventions had significantly better classification than the control group. For the NOVA classification question, the MyPlate + NOVA intervention had significantly better classification than both the MyPlate intervention and the control group (average M = 1.03 compared to 0.15 and 0.24, respectively.)

## 4. Discussion

This study assessed the impact of a simple nutrition education intervention consisting of MyPlate and NOVA principles on knowledge of MyPlate food groups, MyPlate limit status, and NOVA categories. To our knowledge, this is the first experimental study to examine the impact of NOVA principles in this manner and presents a proof-of-concept approach to further develop the field.

Findings indicated that the MyPlate intervention group showed significantly greater improvement in their ability to classify foods into ‘limit’ categories, but not conventional MyPlate food groups, compared to the control group. There was some evidence that food items belonging to more than one MyPlate group, such as Lean Cuisine spaghetti with meat sauce, were more difficult to classify, especially those that would be categorized in the NOVA “ultra-processed” group (not shown). For example, performance for the three combination foods that covered more than one MyPlate group (Lean Cuisine spaghetti with meat sauce, Starbuck’s low-fat blueberry muffin, homemade spinach lasagna) was poor in all groups at baseline and follow-up, averaging 9% and 10%, respectively. In comparison, performance for single-category foods averaged 68% and 70%, respectively. It is possible that prepared, frozen, or fast food meals, which are commonly classified in multiple MyPlate food groups, are more difficult to conceptualize as separate components (e.g., vegetable plus protein plus dairy). Preparing a comparable meal from scratch may be less prone to this type of error if consumers can more easily categorize single ingredients, such as individual vegetables, meats, or cheeses. If this is this case, nutrition education efforts using MyPlate concepts could be less effective.

Participants who received the MyPlate + NOVA group performed better than the control group in classifying foods according to conventional MyPlate food groups, with no differences detected between the MyPlate + NOVA and MyPlate groups. For the ‘limit’ question, both the MyPlate + NOVA and MyPlate groups performed better than the control group. Finally, participants who received the combined MyPlate + NOVA intervention group performed better, on average, than both the MyPlate and control groups in classifying foods by NOVA category.

This was a proof-of-concept study with some notable limitations. For example, classification of the 25 food items was conducted according to the USDA SuperTracker (discontinued as of June 2018). Some food items did not appear exactly as assessed; for example, the Starbuck’s low-fat blueberry muffin utilized nutrient information from a “muffin, blueberry” and McDonald’s French fries utilized information from “French fries, frozen, deep fried (fast food fries)”. It is possible that discrepancies between the varieties may have led to misclassification, but most foods were precise matches, decreasing the possibility of bias.

Some participants likely had existing knowledge of MyPlate and perhaps NOVA, as evidenced by higher baseline performance on the MyPlate questions (61% correct responses overall) compared to the NOVA categories question (35% correct responses overall). At follow-up, means for MyPlate questions were still higher (62%) compared to NOVA (41%). However, increases in performance following the intervention were consistently higher for the MyPlate + NOVA treatment group compared to the MyPlate group, suggesting that NOVA principles may be more easily learned. Participants were college students, a key demographic for nutrition education efforts, but also highly educated, which could have influenced our findings in the positive direction. Finally, participants were instructed to study the intervention, but whether they did is not possible to ascertain with our study design.

This analysis also did not examine behavior change, but nutrition knowledge is suggested to be an important mediating component of dietary habits [30,31,32]. Anthropometric changes were also not assessed given the short period of intervention. The MyPlate tool has not been widely studied as an agent of dietary change. Most studies suggest relatively low reach and use in populations, although some data suggest MyPlate use is associated with improved dietary parameters [33,34,35].

Follow-up survey data were collected immediately after the educational intervention, so our findings do not reflect the possible longer-term effects of such interventions. Longer-term trials are needed to determine the impact of MyPlate and NOVA interventions on dietary patterns and health outcomes across diverse populations. Our results suggest that for common foods, NOVA principles are more easily understood and applied than those of MyPlate in identifying unhealthier highly processed foods. The combined intervention seemed to perform at least as well as the MyPlate only intervention in identifying conventional food groups. These findings present potentially significant opportunities for nutrition education and food literacy programs, in addition to the development of more relevant or more easily applicable dietary guidelines.

## 5. Conclusions

Food and nutrition education interventions utilizing innovative tools such as NOVA that simplify understanding of prevalent and available foods may complement existing food guidelines. Specifically, strategies focusing on level of food processing may be effective in the context of the modern food environment.

## Figures and Tables

**Table 1:** Foods included in survey pre- and post-intervention. MyPlate food groups based on USDA SuperTracker, NOVA categories based on published guidelines.

Food Item	MyPlate Food Groups ^1^ and Limit Status ^2^						NOVA Category ^3^
	Fruit	Veg	Grains	Protein	Dairy	Limit *	
Cooked beef steak, plain				X		fat	1
Olive oil						fat	2
Fruit salad, plain	X						1
Baked Potato, homemade plain		X					1
Vegetable chips, Terra Mediterranean		X					4
Canned mixed vegetables, Del Monte		X				salt	4
Low-fat yogurt, apricot, fruit on bottom, Chobani					X	sugar	4
Granola bar, Chewy Chocolate Chip, Quaker			X			fat, sugar	4
Spinach lasagna, homemade		X	X		X	fat, salt	3
Butter, unsalted						fat	2
Spaghetti with meat sauce, Lean Cuisine		X	X	X	X	salt	4
French fries, McDonald’s		X				fat	4
Peanut butter, Peter Pan				X		fat	4
Blueberry muffin, Starbuck’s			X	X		fat, sugar	4
Canned tomato paste, Del Monte		X					1
Crackers, Triscuit			X			salt	3
Crackers, Wheat Thins			X			salt, sugar	4
Whole grain bread, Ezekiel			X				4
Zucchini bread, homemade			X			sugar	3
Dried apricots, plain	X						1
Plain organic yogurt, Stonyfield					X	fat	1
Mixed nuts, plain				X		fat	1
Black beans, cooked, plain		X		X			1
Salmon, smoked				X		salt	3
Sugar, Turbinado in the Raw						sugar	2

^1^ Based on USDA SuperTracker (www.supertracker.usda.gov); ^2^ Limit status: Saturated fat if exceeded 10% of energy content for food or serving size for food exceeded 10% of the recommended maximum for the day. Sodium (salt) if exceeded 1 mg/kcal. Sugar (sugar) if added sugars exceeded 10% of energy content for food or serving size for food exceeded 10% of recommended daily maximum. (Pan American Health Organization. Pan American Health Organization Nutrient Profile Model. Washington, DC: PAHO, 2016.) ^3^ NOVA categories: 1—Unprocessed/minimally processed, 2—Processed culinary ingredients, 3—Processed foods, 4—Ultra-processed food products. * Indicates specific nutrient/s to limit; X Indicates MyPlate food group to which each food belongs.

**Table 2:** Unadjusted mean proportion (SD) of correct responses averaged over 25 food items at baseline and follow-up, and *p*-value for each of three survey questions, by intervention group.

Survey Question (Q) Item by Intervention Group	Baseline, % Correct (SD)	Follow-Up, % Correct (SD)	% pt. Change (95% CI)	*p*-Value
MyPlate Intervention (*n* = 57)				
Q1: MyPlate food groups	59.9 (11.3)	62.0 (12.2)	+2.2 (−1.3, 5.7)	0.2
Q2: MyPlate limit status	61.7 (11.4)	65.3 (10.7)	**+3.6 (0.6, 6.7)**	0.02
Q3: NOVA categories	34.1 (15.5)	36.6 (14.3)	+2.5 (−0.8,5.9)	0.1
MyPlate + NOVA intervention (*n* = 66)				
Q1: MyPlate food groups	60.7 (12.4)	64.7 (9.4)	**+4.0 (1.0, 7.0)**	0.01
Q2: MyPlate limit status	64.4 (12.7)	70.4 (10.9)	**+6.0 (3.2, 9.0)**	< 0.001
Q3: NOVA categories	34.7 (14.6)	45.8 (15.6)	**+11.1 (6.8, 15.3)**	< 0.001
Control (*n* = 51)				
Q1: MyPlate food groups	63.1 (9.2)	59.8 (12.9)	**−3.2 (−6.0, 0.0)**	0.03
Q2: MyPlate limit status	64.2 (12.6)	61.4 (15.0)	−2.8 (−6.6, 0.9)	0.1
Q3: NOVA categories	36.3 (11.7)	38.8 (14.1)	**+2.5 (0.0, 5.0)**	0.05

*p*-values by paired *t*-test. Bold indicates statistical significance.

**Table 3:** ANOVA *p*-values for each factor. Each ANOVA analyzes a different classification question. *p*-values in bold are statistically significant.

Factor	Classification Question		
	MyPlate	Limit Status	NOVA
Intervention	0.0010	0.0007	<0.0001
Sex	0.5967	0.8745	0.4105
Intervention * Sex	0.6712	0.0246	0.4431
Class	<0.0001	0.7991	0.0016

Classification questions: MyPlate refers to the survey question related to classifying foods according to MyPlate definitions. Limit Status refers to the survey question related to classifying foods as being those to limit due to excess fat, sodium, or sugar. NOVA refers to the survey question related to classifying foods according to NOVA definitions. * Indicates test for interaction.

**Table 4:** Mean McNemar statistics of each intervention group within the ANOVA for each classification question.

Intervention	Classification Question		
	MyPlate	Limit Status	NOVA
MyPlate	0.19 ^ab^	0.47 ^a^	0.15 ^b^
MyPlate + NOVA	0.50 ^a^	0.52 ^a^	1.03 ^a^
Control	−0.51 ^b^	−0.30 ^b^	0.24 ^b^

Different letter superscripts indicate statistically significant differences between treatment groups using Tukey’s pairwise comparisons at the 1% significance level.
